# Emerging Role of Transcutaneous Ultrasound in the Diagnostic of Lung Cancer

**DOI:** 10.3390/cancers17233873

**Published:** 2025-12-02

**Authors:** Corinna Trenker-Burchert, Marius Dohse, Hajo Findeisen, Andreas Schuler, Christian Görg

**Affiliations:** 1 Department of Hematology/Oncology, Marburg University Hospital, Philipps University of Marburg, 35043 Marburg, Germany; 2 Praxis Für Gastroenterologie, DOC-da MVZ GmbH Göppingen, 73033 Göppingen, Germany; 3 Interdisciplinary Center of Ultrasound, Department of Gastroenterology, Marburg University Hospital, Philipps University of Marburg, 35043 Marburg, Germany

**Keywords:** lung cancer, bronchial carcinoma, ultrasound, staging, ultrasound, transcutaneous ultrasound, CEUS, diagnostic imaging, biopsy, clinical workflow

## Abstract

Lung cancer is one of the most commonly diagnosed malignancies worldwide and continues to be a leading cause of cancer-related mortality. Precise staging is crucial for predicting outcomes and directing treatment decisions. Current international guidelines mainly recommend imaging techniques like CT and PET-CT, with limited references to transcutaneous ultrasound, which is only suggested in particular clinical cases. Ultrasound provides real-time imaging, high resolution in near-field structures, and the ability to assess thoracic wall infiltration, supraclavicular and cervical lymph nodes, pleural effusions, and metastatic lesions. Furthermore, ultrasound-guided biopsies can enable quick and safe histological confirmation of accessible lesions. Based on these advantages and a review of current literature, we propose that integrating ultrasound into staging algorithms could improve diagnostic efficiency, decrease invasive procedures, and support prompt treatment planning. We also highlight the need for further research in this area.

## Simple Summary

Lung cancer is one of the most common and deadly cancers worldwide. Accurate histological confirmation and staging are essential in clinical care. While CT and PET-CT are established standards in the staging process, the potential of ultrasound as a real-time, cost-effective tool may not yet be fully recognized in lung cancer staging. This review discusses how ultrasound, particularly when combined with contrast-enhanced ultrasound, can aid in detecting tumor spread, guide safe biopsies, and reduce the need for more invasive procedures. By highlighting current evidence and future research directions, we aim to show how the implementation of sonography could improve the diagnosis and staging of lung cancer.

## 1. Introduction

Worldwide, lung cancer is considered to be the most diagnosed malignant disease, with approximately 2.5 million new cases in 2022 and the highest number of cancer-related deaths [1]. In Germany, small cell lung cancer (SCLC) accounts for about 12–15% of lung cancer cases, while non-small cell lung cancer (NSCLC) comprises approximately 85%. Among NSCLC cases, adenocarcinoma is the most prevalent subtype, occurring in 40% of men and 48% of women [2,3,4,5]. Notably, 77–79% of NSCLC cases and up to 95% of SCLC cases are diagnosed at an advanced or metastatic stage (Union internationale contrele cancer (UICC) stage III-IV) [3,4,6,7,8,9].

According to the German AWMF (Arbeitsgemeinschaft der Wissenschaftlichen Medizinischen Fachgesellschaften) S3 guidelines and international guidelines (the European Society for Medical Oncology (ESMO) and the American Society for Clinical Oncology (ASCO)), chest computer tomography (CT) and positronen emissions tomographie (PET-CT) are considered the standard diagnostic tools for staging bronchial carcinoma [2,10,11].

Chest ultrasound has become an established diagnostic technique characterized by high sensitivity and specificity in the identification of thoracic wall lesions, pneumonia, pneumothorax, pulmonary embolism, and pleural effusion [12,13,14,15,16]. Advanced sonographic diagnostic approaches, including color Doppler ultrasound, contrast-enhanced ultrasound (CEUS), and ultrasound-guided interventions, are also applicable to thoracic pathologies. Nevertheless, despite extensive scientific evidence, their translation into clinical routine remains limited. Key limitations include restricted operator expertise and considerable interobserver variability. In addition, transcutaneous thoracic ultrasound is physically limited by the total reflection of ultrasound waves at osseous structures and by the interference caused by intrapulmonary air [5,17]. Notably, non-small cell lung cancers (NSCLCs) predominantly manifest in peripheral regions. A review of pN1 NSCLC cases showed that 222 of 324 (69%) had tumors at the periphery, with 169 of these (76%) showing pleural invasion [18], making them potentially suitable for sonographic diagnosis.

According to the German AWMF-S3 guidelines for NSCLC and SCLC, transcutaneous ultrasound is used only for selected clinical indications [2,11,19,20]. According to comprehensive international lung cancer guidelines (ESMO, ASCO), transcutaneous ultrasound has little or no role in disease staging (see Table 1) [2,11,19,20]. Due to the high resolution of high-frequency ultrasound in the near-field area and its ability to provide real-time imaging, ultrasound can offer valuable information for diagnosing pathology in the thoracic wall and neck [21]. The purpose of this manuscript is therefore to discuss the potential of transcutaneous ultrasound in the diagnosis and staging of lung cancer.

## 2. Transcutaneous Ultrasound in the Primary Staging of Lung Cancer

The staging of lung cancer is currently based on the 9th edition of the TNM classification developed by the International Association for the Study of Lung Cancer, which is used for prognosis assessment and treatment decision-making [2,11,19,20]. This classification evaluates tumor size at the primary site (T), regional lymph node involvement (N), and distant metastasis (M). The following section discusses how ultrasound is used to evaluate the T, N, and M stages [2,11,19,20].

## 3. Transthoracic Ultrasound in the Evaluation and Characterizing of Local Tumor Size (T-Stage)

**Figure 1:**
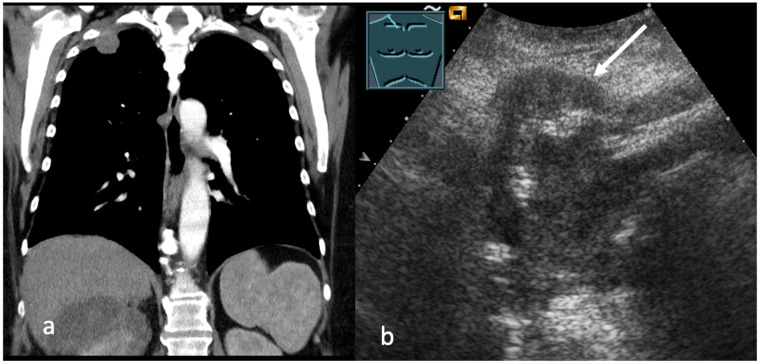
(**a**,**b**) Lung cancer in CT (**a**). B-US presents a tumor with complex echogenicity (**b**) and with infiltration of the thoracic wall (arrow).

Due to limited comprehensive imaging, the use of transthoracic ultrasound in determining local tumor extent (T-stage) is restricted, and CT imaging remains essential [2]. Currently, the ESMO and ASCO guidelines do not recommend transthoracic ultrasound for T-stage assessment (Table 1) [2,11,19,20,22]. In the German AWMF-S3 guidelines, chest ultrasound is advised alongside CT to evaluate thoracic wall infiltration [2,11,19,20]. Compared to CT, which has a sensitivity of 68% and a specificity of 66% for detecting thoracic infiltration [5,23,24], transthoracic ultrasound is suitable for the assessment of this issue [5,23,24]. Real-time ultrasound can be used to assess whether a pleural-based mass moves synchronously with respiration (“sliding of the pleural lesion”) or remains fixed— representing one of three key criteria for evaluating thoracic wall infiltration. [24]. An interruption of the pleural line and tumor infiltration into the thoracic wall are additional criteria for assessing infiltration [24]. Applying these three criteria allows chest wall infiltration to be detected with a sensitivity of 100% and a specificity of 98% [24]. Other studies, such as those by Caroli et al., report similar findings, detecting chest wall infiltration with a sensitivity of 89%, a specificity of 100%, and a positive predictive value of 100% [25,26]. Thoracic wall infiltration occurs in <10% of patients diagnosed with bronchial carcinoma [24,27,28]. Since the detection of thoracic wall infiltration is clinically significant, sonography may serve as a crucial modality in this process [2,11,19,20]. Thoracic wall infiltration influences the type and extent of surgery [29]. An infiltration usually requires a more extensive operation, such as a chest wall resection [29]. The use of contrast-enhanced ultrasound (CEUS) for lung lesions enables differentiation between bronchial artery (BA) and pulmonary artery (PA) vascularization. Notably, malignant tumors display BA vascularization in 72% of cases [30]. With CEUS, central lung tumors in obstructive atelectasis are more clearly visualized (92.6% vs. 75.9% without CEUS) [31]. Additionally, ultrasound is generally considered superior to CT in assessing peritumoral atelectasis (*p* = 0. 017) [32]. Figure 1 presents lung cancer with infiltration of the thoracic wall.

## 4. Transcutaneous Ultrasound in the Evaluation of Supraclavicular and Collar Lymph Nodes

In the AWMF-S3 guidelines, as well as the ESMO (European Society for Medical Oncology) and ASCO guidelines, transcutaneous ultrasound has no relevance in the evaluation of lymph nodes (N-stage) [2,11,19,20].

Based on the International Association for the Study of Lung Cancer’s (IASLC) classification of regional lymph node stations, only lymph nodes in the supraclavicular and deep cervical regions, up to the level of the thyroid cartilage, are considered part of the regional thoracic lymph node metastasis (N3) stage [33]. Lymph nodes above the thyroid cartilage indicate distant metastasis (M1) [33].

Because transcutaneous ultrasound has limitations in the thorax, the assessment of hilar lymph nodes (N1 or N2 status) is not feasible [2,3,4,5]. However, this is the role of endobronchial ultrasound (EBUS) and endobronchial interventions (EUS), which are also recommended in the guidelines [2,11,19,20]. Transcutaneous ultrasound, however, shows significant advantages (*p* = 0.002) over CT in evaluating supraclavicular and cervical lymph nodes (N3) [32]. CT detects pathological cervical lymph nodes in solid tumors with a sensitivity of 85%, specificity of 86%, and diagnostic accuracy of 85%, making it less effective than ultrasound evaluation, which shows a sensitivity of 90%, specificity of 90%, and diagnostic accuracy of 90% [34,35]. Prosch et al., based on a prospective study, defined malignant criteria for supraclavicular lymph nodes in ultrasound as the absence of the hilum sign, a short-axis diameter of ≥5 mm, and a round shape [28]. Additional suspicious features for malignancy include indistinct borders, calcifications, necrotic areas, and peripheral or mixed vascularization [28,36]. Ultrasound has the benefit of being a clinical examination method that allows for the use of clinical criteria to assess malignancy, in addition to imaging criteria, which are important in deciding whether to perform a biopsy. A study by Vassilakopoulos et al. identified six key clinical factors (age, tenderness, size, itching, supraclavicular lymphadenopathy, and echotexture). By using a scoring system, they found a sensitivity of 95.2% (95% confidence interval [CI]: 88.1–98.1%) and a specificity of 81.0% (95% CI: 75.4–85.6%) to predict whether histological confirmation was needed [37].

## 5. Transcutaneous Ultrasound in the Evaluation Distant Metastasis

The detection of distant metastases (UICC stage IV) at diagnosis guides therapy decisions [2,11,19,20]. Newly diagnosed NSCLC and SCLC cases are likely to have a high prevalence of distant metastases, making timely confirmation an important task in real-world medicine. According to an analysis of distant metastasis patterns among lung cancer subtypes, osseous metastasis was the most common synchronous site in adenocarcinomas (35.1%), followed by cerebral metastases (26.5%), lung (25.2%), and liver (13.2%) [38]. The adrenal glands are also frequently affected (15% in NSCLC; 10% in SCLC), although the reported frequencies vary in meta-analyses [39]. However, in 39.5% of cases, multiple sites of synchronous distant metastases are present. In other subtypes of NSCLC, such as large cell neuroendocrine carcinoma, the liver is more frequently affected (26.4%) [38]. In SCLC, the liver is the most common site of synchronous metastasis (35.4%) [38]. Additionally, in 42.3% of SCLC cases, multiple sites of synchronous distant metastases are present [38].

Ultrasound-guided biopsy for histological confirmation of liver metastases or malignant pleural effusion may help facilitate faster therapy decisions. More invasive diagnostic procedures such as EBUS for assessing hilar lymph nodes as routine part of staging may be reduced in these cases. This can spare patients unnecessary diagnostic procedures with potential complications, reduce costs associated with diagnostics that have no therapeutic impact, and shorten the pre-therapeutic phase [17]. A prospective study on implementing ultrasound in lung cancer staging to address these questions is warranted.

According to ESMO and ASCO guidelines, ultrasound has no role in detecting distant metastases [2,11,19,20]. In contrast, the AWMF S3 guidelines recommend ultrasound for detecting pleural effusion [2,11,19,20].

A malignant pleural effusion is defined as a distant metastasis (M1) in lung cancer [2,11,19,20].

**Figure 2:**
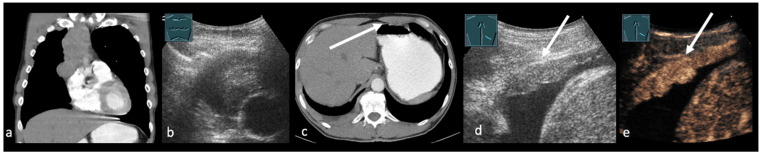
(**a**–**e**): Central lung cancer in a CT scan (**a**). B-US presents a hypoechoic lung tumor in the upper mediastinum (**b**). In addition, the CT scan shows a suspected pleural metastasis (arrow) (**c**), which appears as a hyperechoic, nodular structure on B-US (arrow) (**d**). CEUS (**e**) shows homogenous contrast enhancement of the lesion (arrow), suggestive of pleural carcinomatosis.

Ultrasound, with a sensitivity of 94% and a specificity of 98%, surpasses chest X-ray (which has a sensitivity of 51% and specificity of 91%) and CT scans in detecting pleural effusion, making it the preferred method for this purpose [32,40]. Ultrasound is capable of diagnosing and guiding punctures for fluid volumes as small as 5 milliliters [41]. Cytological confirmation of the effusion is crucial because, aside from pleural tumors, no other diagnostic criteria—such as echogenicity, septation, pleural thickening, or CEUS patterns—are specific for malignancy [42]. However, in patients with suspected malignant pleural effusion, CEUS can increase sensitivity from 69.2% to 92.3% and specificity from 63% to 90% [43]. In this analysis, lung consolidation showing inhomogeneous enhancement was associated with malignancy (*p* < 0.05) [43], and malignant pleural lesions were significantly more likely to be detected with CEUS [44]. Figure 2 presents a central lung tumor with pleural carcinomatosis.

Involved lymph nodes above the thyroid cartilage [33] are defined as distant metastasis, which indicates an M1 situation, corresponding to UICC Stage IV in lung cancer. Therefore, neck ultrasound should be performed as the diagnostic method with the highest sensitivity and specificity (see chapter N-stage and interventions) for confirming distant metastasis in this area. In general, cost-effective B-US, despite the lack of specific recommendations in guidelines, may often be the first imaging modality for detecting abdominal metastases due to its rapid availability and absence of radiation exposure in clinical practice [45]. However, the sensitivity of 55% of B-US for detecting liver metastases, when used as the sole imaging method in staging diagnosis, is low [46], making a recommendation in guidelines unreasonable. CEUS improves the sensitivity and specificity of B-US in diagnosing liver metastases and allows for the characterization of unclear liver lesions [47,48]. A multicenter study found a diagnostic advantage (*p* = 0.0006) of CEUS over B-US in detecting hepatic metastases in patients with colorectal carcinoma, suggesting that CEUS could also be significant in detecting liver lesions in other tumor types [5,49]. In a retrospective analysis, synchronous liver lesions in patients with lung cancer were malignant in 53.9% of cases. Synchronous liver lesions cannot be reliably identified with B-US, especially in tumor patients. CEUS enables the characterization of focal liver lesions with a sensitivity of 93% and a specificity of 90% [50]. CEUS is also recommended for further differentiation of focal lesions when CT or MRI findings are unclear [51]. Therefore, sonographic liver staging in patients with lung cancer relying only on B-US without CEUS is limited.

## 6. Ultrasound Guided Biopsy

Molecular therapeutic stratification and immunological features of tumors have gained significant importance in recent years [2,11,19,20] and are part of the standard diagnostic procedures proposed by guidelines [2,11,19,20]. As a result, adequate histological sampling has become a key aspect of lung cancer diagnostics [5]. Common options for histological sampling include ultrasound-guided transcutaneous or CT-guided biopsies, along with endoscopic procedures such as bronchoscopy and EBUS. In a combined meta-analysis, sample collection using endobronchial ultrasound transbronchial needle aspiration (EBUS-TBNA) was sufficient for next-generation sequencing in 86.5% of cases, meaning that in about 13–15%, a new sample is needed [52]. Currently, only the German AWMF-S3 guideline recommends ultrasound-guided histological sampling of thoracic wall lesions (Table 1) [2,11,19,20].

The ESMO guidelines advise “imaging-guided transthoracic, percutaneous biopsy of peripheral lesions,” emphasizing that “typically, CT is proposed” [2,11,19,20]. If the tumor is visible via ultrasound in the thoracic region, or in cases of suspicious lymph nodes or abdominal metastasis-related lesions (e.g., liver or adrenal glands), an ultrasound-guided biopsy is a very feasible alternative. Transhepatic biopsies of adrenal glands or deep abdominal metastases are also feasible [53]. Due to the known benefits of ultrasound interventions—including quick availability, cost-effectiveness, real-time imaging, and absence of radiation exposure—this approach is an attractive option [5]. Ultrasound-guided interventions, along with pre- and post-interventional management, should be performed according to the guidelines of the EFSUMB (European Federation of Societies for Ultrasound in Medicine and Biology) [5,54]. By using full-cutting core needles (18 Gauge), 30–59% more tissue can be obtained compared to core biopsy needles without full-cutting cylinders [55,56]. Since larger tissue samples are needed for molecular diagnostics (NGS) and insufficient material is obtained by EBUS-TBA in about 10–13% of cases [52], using transcutaneous full-thickness biopsy for both liver and lung biopsies seems reasonable, as long as it is accessible for ultrasound-guided biopsy. While ultrasound-guided biopsy for confirming liver and lymph node metastases is a well-established method, the use of ultrasound-guided transcutaneous thoracic biopsies is less common. In one study, the diagnostic confirmation of thoracic core biopsies achieved a sensitivity of 89%, a diagnostic specificity of 97%, and a diagnostic accuracy of 93% [5,57,58].

The complication rates of thoracic interventions, based on a meta-analysis of 12 studies, were generally 3.6% (including 0.03% major bleeding complications) [59]. The complication rates for ultrasound-guided transthoracic biopsies, reported as 3% in another analysis, were significantly (*p* < 0.001) lower compared to CT-guided biopsies, which had a rate of 24.3% [24,60,61]. However, it is important to note that ultrasound-guided biopsies can only target lesions that contact the pleura [5,57]. After peripheral lymph node biopsies, major complications occurred in 0.1% of cases [62], and in liver biopsies, major complications occurred in 0.4% of cases [63]. Using CEUS before histological confirmation allows for significantly more frequent detection of necrosis in thoracic lesion (40.7% vs. 16.7%; *p*= 0.003) [5,64]. This enables targeting biopsies to the vital areas of the tumor. The success rate of thoracic biopsies was significantly higher when CEUS was used (96.3%) compared to ultrasound-guided biopsies without CEUS (80.3%) (*p* = 0.008) [5,64]. Applying CEUS also resulted in a change in the initially planned puncture site in 48.1% of cases, highlighting its added benefit in improving biopsy accuracy and targeting [5,64]. In Hafez et al.’s analysis of the use of ultrasound in diagnosing lung cancer, histological confirmation was achieved in 78% of cases with ultrasound-guided biopsy [32].

**Figure 3:**
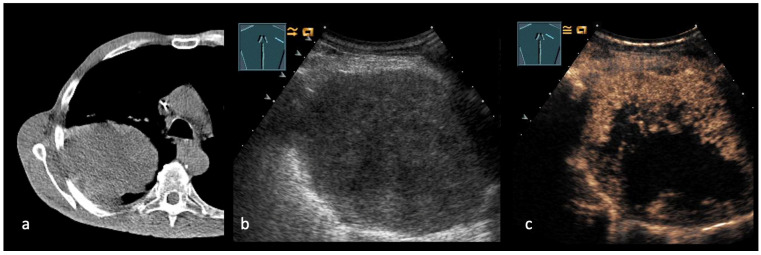
(**a**–**c**): CT-scan of a lung cancer (**a**). B-US presents a round, hypoechoic pulmonal lesion (**b**) with a large central, necrotic area in CEUS (**c**).

**Figure 4:**
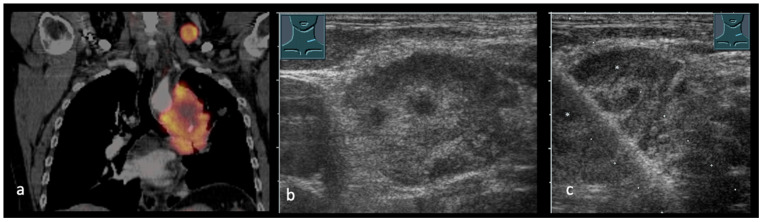
(**a**–**c**): Central lung cancer and suspected supraclavicular lymph node metastasis in PET-CT-scan (**a**). B-US presents a supraclavicular lymph node with complex echogenicity with tracer accumulation in PET-CT (**b**). Ultrasound guided biopsy of the suspected lymph node (**c**).

However, the study did not use CEUS for necrosis detection before biopsy, and necrotic material was a major cause of failure in histological confirmation [32]. The results from Liang et al. suggest that using CEUS prior to biopsy could increase diagnostic yield, and this hypothesis warrants validation through prospective studies [64]. Figure 3 presents lung cancer with large, central necrosis, which again clarifies the necessity of CEUS before biopsy. In addition to biopsies of pleural-based tumors, ultrasound-guided biopsies are also possible for tumor formations in atelectasis [65,66]. With the additional use of CEUS, Lei et al. achieved a biopsy success rate of 98%. In 6 out of 112 patients, complications such as hemoptysis occurred during the biopsy, and 10 out of 112 patients developed bloody sputum after the procedure [66]. Moreover, it is important to highlight the special case of the pancoast tumor located at the apex of the lung (5% of lung carcinoma), which can only be accessed endoscopically in 10% of cases [67,68,69,70,71]. Pancoast tumors are located at the apex of the lung and tend to grow aggressively into the chest wall (infiltration of the parietal pleura), nerves (e.g., brachial plexus), and blood vessels, producing typical clinical signs such as pain, paresthesia, or other neurological dysfunctions [72]. Due to their location, Pancoast tumors are well visualized via transcutaneous ultrasound [67]. In conventional chest X-ray, Pancoast tumors are not always detectable [73]. Clinically, patients often present with unexplained shoulder pain due to the tumor’s infiltration into the neuroforamina [72,73]. A tumor involvement of the stellate ganglion causes the well-known Horner syndrome (ptosis, miosis, and anhidrosis) [72]. Therefore, ultrasound of the lung apex should be recommended when this symptom appears [5,73]. There is only one case series on the role of ultrasound in Pancoast tumors [67]. In this series, the tumors were visible in all cases and confirmed histologically in 100% of [67]. It is also worth noting the crucial role of ultrasound in correlating findings from CT/PET-CT, as well as using ultrasound-guided biopsy to verify these results histologically. A representative meta-analysis found that PET-CT has a sensitivity and specificity of up to 95% in detecting distant metastases [74,75]; histological confirmation is still essential when making treatment decisions. Solitary extrapulmonary false PET-positive lesions were observed in 9% in patients with lung cancer [76], which further highlights the clinical relevance of histological confirmation. Figure 4 shows a case of a PET-positive lesion, along with the sonographic correlate and the sampling procedure, demonstrating the supporting role of ultrasound in such cases.

## 7. Discussion

**Figure 5:**
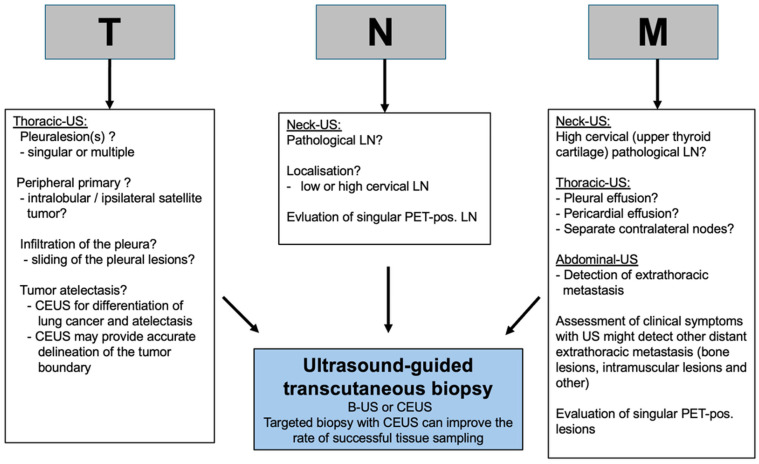
Strategic proposal for implementing sonography in the staging diagnostic of lung cancer. T: tumor size; N: nodus; M: metastasis; LN: lymph node; US: ultrasound; CEUS: contrast-enhanced ultrasound.

Although transcutaneous/transthoracic ultrasound is commonly used clinically, it holds little to no significance in international guidelines, such as those from ESMO and ASCO [2,11,19,20]. Typically, CT and PET-CT scans are the primary tools for diagnosing LC, yielding results with great accuracy for most patients. However, like all methods, they have their own limitations. Therefore, we aimed to review the literature on how ultrasound can provide additional information for accurate and therapy-relevant staging, and to propose a strategy for incorporating ultrasound into clinical workflows as a reference for future studies. Figure 5 summarizes the indications for using transcutaneous/transthoracic ultrasound in lung cancer staging, based on our review. Fast and reliable tissue sampling is absolutely crucial, especially when it needs to be sufficient for large-scale molecular analysis. Considering the benefits of ultrasound mentioned above, it can play a key role in diagnosing and staging of lung cancer, especially when combined with CEUS. Hafez et al. systematically evaluated ultrasound in the staging process alongside standard CT diagnostics and found ultrasound to be significantly better than CT at detecting peritumoral atelectasis, diaphragm paralysis, and supraclavicular lymph node invasion [32], demonstrating the potential for accurate staging according to the TNM system. Multiple studies have demonstrated that ultrasound, as a dynamic imaging technique, is superior to other methods for assessing thoracic wall infiltration, leading to a recommendation in German guidelines [5,11,19,20,23,24]. Studies have demonstrated that CEUS is more effective for accurate tumor delineation and targeted biopsy of viable regions [43,64] (Figure 5).

Delayed diagnosis due to slow processes is likely to be adverse for the patient. A practical approach could be to use neck/thoracic and abdominal ultrasound during the early staging process, especially when other imaging methods are not readily available (Figure 5). If the tumor and/or metastatic lesion are clearly visible, therapeutic decisions might be made more quickly; in advanced cases, some interventions or procedures may not be necessary.

Despite the emerging evidence of its potential role, limited data are available on the impact of ultrasound as an additional diagnostic tool for therapy-relevant outcomes, survival, and cost-effectiveness in lung cancer staging diagnostics, highlighting the need for further research in this area. Despite the emerging evidence of its potential role, limited data are available on the impact of ultrasound as an additional diagnostic tool for therapy-relevant outcomes, survival, and cost-effectiveness in lung cancer staging diagnostics. Additionally, artificial intelligence (AI)-assisted ultrasound is a new field in the diagnosis of thoracic pathologies [17]. Initial reports demonstrate the use of AI in assessing B-lines, pulmonary edema, and pneumothorax [77,78,79], underscoring the need for further research concerning malignant alterations, such as pleural invasion.

## 8. Summary

Based on a review of existing literature, we propose a potential ultrasound first-approach for the implementation of sonography in the diagnosis and staging of lung carcinoma.

## Figures and Tables

**Table 1:** Transcutaneous ultrasound in the AWMF (Arbeitsgemeinschaft der Wissenschaftlichen Medizinischen Fachgesellschaften), European Society for Medical Oncology (ESMO), and American Society for Clinical Oncology (ASCO) guidelines for lung cancer [2,11,19,20].

	Ultrasound in the Diagnostic of Lung Cancer
AWMF	Detection of pleural effusion Assessment of thoracic wall infiltration Ultrasound-guided biopsy for tumors located in the chest wall Additional contrast-enhanced ultrasound of the liver in addition to FDG-PET CT and MRI if a solitary liver metastasis is suspected
ESMO	Imaging guided transthoracic, percutaneous biopsy of peripheral lesions (typically CT is proposed)
ASCO	No recommendations
